# Potential Targets for Noninvasive Brain Stimulation on Depersonalization-Derealization Disorder

**DOI:** 10.3390/brainsci12081112

**Published:** 2022-08-21

**Authors:** Sisi Zheng, Nan Song, Sici Wang, Yanzhe Ning, Hong Zhu, Mingkang Song, Yuan Jia, Hongxiao Jia

**Affiliations:** 1 The National Clinical Research Center for Mental Disorders & Beijing Key Laboratory of Mental Disorders, Beijing Anding Hospital, Capital Medical University, Beijing 100088, China; 2 Advanced Innovation Center for Human Brain Protection, Capital Medical University, Beijing 100088, China

**Keywords:** functional connectivity, non-invasive brain stimulation, depersonalization-derealization disorder, transcranial magnetic stimulation, functional magnetic resonance imaging

## Abstract

Introduction: Non-invasive brain stimulation seems to be beneficial for DPD patients. However, the sites used in previous studies were empirical. Exploring new stimulation locations via functional magnetic resonance imaging may improve the efficacy. Objectives: The objective was to find potential locations for non-invasive brain stimulation on the depersonalization-derealization disorder. Methods: We explored the potential brain surface regions from three pipelines: pipeline 1: activation likelihood estimation meta-analysis (five studies with 36 foci included); pipeline 2: functional connectivity analysis based on DPD-network (76 subjects included); and pipeline 3: functional connectivity analysis based on DPD regions of interest from the meta-analysis. Potential targets were the 10–20 system coordinates for brain surface regions. Results: We identified several potential brain surface regions, including the bilateral medial prefrontal cortex, dorsal lateral prefrontal cortex, superior parietal gyrus, superior temporal gyrus, and right ventrolateral prefrontal cortex as potential sites. Conclusion: Our findings of the potential stimulation targets might help clinicians optimize the application of non-invasive brain stimulation therapy in individuals with DPD.

## 1. Introduction

Depersonalization-derealization disorder (DPD) or depersonalization-derealization syndrome is characterized by the experience of detaching from oneself or detaching from one’s surroundings [1]. Symptoms usually first appear in adolescence, especially when the individual is 15–19 years old [2]. Teenagers who reported feeling detached or unreal would be ignored by their parents due to their low awareness of the disease. This added more pain to the situation. In addition, data showed that DPD can be underdiagnosed, resulting in patients waiting 7 to 12 years until they receive a diagnosis [3]. Despite that, it seems that the prevalence rates of DPD are not low, which have been reported as about 1–2% [4,5], even reaching up to 19.1% in a Canadian survey [6]. The high incidence is accompanied by injury to the life and work of the patient, leading to heavy social costs. Researchers found that depersonalized individuals have difficulties with cognitive processes, particularly in early perceptions and attention [7,8]. Moreover, depersonalization was reported as an independent risk factor for the persistence or elevation of depressive/anxiety symptoms (contributing to suicidal ideation) [9] as an easily assessed risk factor for the course of a mental illness disorder [10].

DPD treatment has changed from operative therapy to pharmacotherapies, psychotherapies, and physiotherapies as a result of the advancements in psychology and psychopathology. Several systematic reviews concentrating on the treatment of DPD have been reported, containing pharmacotherapy and transcranial magnetic stimulation (TMS) [11]. Depersonalization is predominantly treated with drugs such as lamotrigine, psychological treatment such as cognitive behavior therapy [12], or physiotherapies such as repetitive TMS (rTMS) [13]. However, empirically validated treatments for DPD remain scarce.

Non-invasive brain stimulation (NIBS) could be an approach for treating DPD. Recently, NIBS techniques—such as rTMS and transcranial direct current stimulation (tDCS)—have gathered substantial interest in the clinical practice and research of psychiatric disorders. Furthermore, NIBS techniques seem to be effective in treating treatment-resistant mental disorders. CANMAT guidelines [14] recommend rTMS as a first-line intervention after one adequate antidepressant trial fails. The effectiveness of both rTMS and tDCS in reducing negative symptoms in schizophrenia was superior to that of sham stimulation according to a systematic review [15]. Refractory obsessive-compulsive disorder may also benefit from different TMS or tDCS [16]. What is more, previous studies have shown the benefit of TMS on DPD [11]. Thus, treatment of DPD may be possible with NIBS techniques. However, the sites used in the previous studies were empirical. Novel targets of NIBS still could be explored to improve the efficacy of NIBS in treating DPD patients.

NIBS brain surface location could be identified using a combination of meta-analysis and functional connectivity (FC) analysis. According to Zhang et al. [17], several potential locations for NIBS in depressive disorder patients could be found from seed-based FC analysis, while the regions of interest (ROI) were identified from 395 functional magnetic resonance imaging (fMRI) studies. Zhang et al. conducted a meta-analysis of 395 fMRI studies to identify depressive disorder-associated brain regions as regions of interest (ROI). This method comprehensively considered the meta-analysis (directly involved in the pathophysiology of disease, FC analysis with the disease network (possessing the strongest correlations with the disease network)), and FC analysis with the disease ROIs (correlated with the largest number of disease ROIs). Additionally, they found that the location of dorsolateral prefrontal cortex (DLPFC) might be located posterior to the coordinates used in most tDCS studies treating depressive disorder (F3, F4, F8, and Fp2). As such, this method may serve as a basis for future clinical investigations of NIBS locations. It could also be successful in identifying potential targets in diseases like schizophrenia [18], autism spectrum disorders [19], and dementia [20]. Based on recent fMRI research, it is possible to explore the NIBS sites involved in DPD using the above methods.

In this study, we aimed to find potential locations for NIBS techniques on DPD and provide a basis for future NIBS clinical research or practices. So, we conducted a meta-analysis and FC analysis with three pipelines to identify potential brain surface and precise location with 10–20 systems.

## 2. Materials and Methods

### 2.1. Participants

#### 2.1.1. Participants and Study Design

This study was approved by the Ethics Committee of Beijing Anding Hospital, Capital Medical University, China, and all protocols were carried out under the guidance of the Declaration of Helsinki. DPD patients (86 subjects enrolled while 76 analyzed) were diagnosed by two trained senior title psychiatrists according to ICD-10 criteria. They were also screened through the Chinese version of the Dissociative Disorders Interview Schedule (DDIS, https://www.rossinst.com/ddis, accessed on 5 September 2018 [21] and the mini-international neuropsychiatric interview (M.I.N.I.) [22]. The M.I.N.I was used to exclude other psychiatric comorbidities. The DDIS was used to determine the existence of DPD and exclude other types of dissociative disorder, borderline personality disorder, schizophrenia, or substance abuse.

#### 2.1.2. Assessment

We used the Cambridge Depersonalization Scale (CDS) [23] to assess patients. CDS is a 29-item self-reporting scale that is used most frequently in recent NIBS clinical trials [13,24,25]. Each item contains two questions: frequency (0~4) and duration (1~6). A larger score means the symptoms are more severe. The maximum score of CDS is 290; the cut-off score is often set as 70 [26,27]. CDS factor analyses have shown that the scale has multiple dimensions, with studies reporting on two [28], four [26,29], and five factors [27]. Regrettably, there is still a lack of factor analyses in the Chinese population. Thus, we chose the five factors version to describe more detail of participants’ characters. It contains “Numbing”, “Unreality of Self”, “Perceptual alterations”, “Unreality of surroundings”, and “Temporal disintegration”.

#### 2.1.3. Inclusion and Exclusion Criteria

The inclusion criteria were as follows: participants who were (a) diagnosed as DPD patients; (b)15–45 years old; (c) Right-handed; (d) with a score of CDS ≥ 70.

The exclusion criteria were as follows: Patients with (a) transient experiences of depersonalization and/or derealization due to trauma, fatigue, or substance use; (b) with other psychiatric comorbidities; (c) a history of neurological disorders or family history of hereditary neurological disorders; (d) history of substance addiction or brain trauma; (e) gross morphological anomalies, as evidenced by brain MRI; and (f) any electronic or metal implants.

### 2.2. MRI Data Acquisition

Rest state Functional Magnetic Resonance Imaging (rs-fMRI) scanner (Prisma 3.0, Siemens, Munich, Germany) 3T was used to collect image data at Beijing Anding Hospital, Capital Medical University, China. Rs-fMRI (with a single-shot, gradient-recalled echo-planar imaging sequence) and high-resolution brain structural images (with a T1-weighted three-dimensional multi-echo magnetization-prepared rapid gradient-echo sequence) were acquired. The detailed parameters are described in the supplementary materials Section 2.2.

The state of participants when scanning, head motion, and previous lifestyles be potential confounders. Thus, the participants were seated in the waiting rooms for a half-hour rest before scanning to prevent falling asleep, and told to keep their eyes closed during the scanning. To minimize head movement during scanning, foam head holders were used. All participants did not drink or smoke before scanning.

### 2.3. FC Analysis

After image preprocessing with the default pipeline in the Data Processing and Analysis for Brain Imaging (DPABI) version 5.1 (http://rfmri.org/dpabi, accessed on 15 April 2022) [30] and SPM12 [31](detailed information see in supplementary materials Section 2.3), we conducted seed-based FC analysis. Briefly, the residual BOLD time course was extracted from the ROIs, and Pearson’s correlation coefficients were computed between ROIs and all other brain voxels. And the coefficients were subsequently transformed into z-scores to increase normality.

Two types of seeds were used in this study: DPD-ROIs and DPD-network. We created 6-mm sphere ROI masks that used the coordinates from the meta-analysis results (toolbox: Marsbar). All the masks were combined to make a DPD-network mask. Thus, we got the first-level maps.

Then, one sample *t*-test was used to perform a second-level analysis with a voxel-wise level threshold of *p* < 0.001 and a cluster-level false discovery rate (FDR) of *p* < 0.05 applied.

Finally, we conducted a sensitivity analysis that we performed in pipeline 2 and pipeline 3 with first-episode DPD patients.

### 2.4. Sample Size Estimation

Sample size was determined by G*power 3.1.9.7 with the parameters as follows: Test family = *t* tests; Statistical test = Means: Difference from constant (one sample case); Tails = two; Effect size = 0.5 (i.e., medium effect); α = 0.05; Power = 0.80. The total sample size was estimated as 34.

### 2.5. Identifying Potential Locations for NIBS

We identified potential locations for NIBS on DPD from three pipelines as follows.

#### 2.5.1. Pipeline 1 Identified Potential Targets from the Meta-Analysis

We conducted an activation likelihood estimation (ALE) meta-analysis to find potential targets (following PRISMA 2020 statement [32]). ALE evaluates the overlap between foci by modeling them as probability distributions centered at the respective coordinates. In ALE, a coordinate-based meta-analysis of neuroimaging results is conducted objectively and quantitatively. The method has been validated for numerous applications [33,34,35].

We searched 7 databases, selected the DPD-related fMRI studies, extracted the coordinates, and performed an ALE meta-analysis by GingerALE [36]. The detailed steps are in the Supplementary Materials Section 1. Thus, we got the coordinates and meta-analysis map. The coordinates reported in Talairach space were converted into Montreal Neurological Institute (MNI) space to conduct the following steps. We selected the brain surface using the mask (Supplementary Materials Figure S1) created in the previous studies [17,18,19]. The brain regions extracted in this pipeline may be directly involved in the pathophysiology of DPD.

#### 2.5.2. Pipeline 2 Identified Potential Targets from DPD-Network-Based FC Analysis

We then performed a DPD-network-based FC analysis (first-level analysis). The second-level correlation map was then filtered to remove clusters smaller than 50 voxels. Four to six surface clusters with the largest peak z-scores were finally selected, with positive and negative correlation maps. These clusters represent the brain surface regions possessing the strongest correlations with the DPD network.

#### 2.5.3. Pipeline 3 Identified Potential Targets from DPD-ROIs Based FC Analysis

The second-level correlation map (created by the similar steps in pipeline 2) of each DPD-associated ROI was saved to a binary mask. The binary masks of all ROIs formed a third-level map (positive and negative correlation maps, respectively). The intensity of each voxel in the third-level map represents the number of DPD–ROIs correlated with the voxel. Finally, we identified four to six surface clusters as potential regions with the largest peak z-scores among all clusters larger than 20 voxels. These clusters represented the brain surface regions that were correlated with the largest number of DPD-ROIs.

#### 2.5.4. Visualization

The results of the three pipelines were mapped onto a standard brain and a standard head with the international 10–20 system in MNI space using Surf Ice (www.nitrc.org/projects/surfice/, accessed on 11 November 2020) and MRIcroGL.

## 3. Results

### 3.1. Meta-Analysis Results

Five studies (number of foci: 36) were included in the meta-analysis section (detailed in the Supplementary Material Table S1). The results of the ALE analysis are shown in Table 1. The activation areas contained the left cingulate gyrus (CG), medial frontal gyrus (mFG), and anterior cingulate.

### 3.2. Potential Targets Identified from Three Pipelines

In total, 84 patients with DPD were enrolled in this study, but eight image data were excluded (one for gross morphological anomalies; three for poor quality control score, three for max head motion > 3 mm, and one for relative RMS > 0.2 mm). Thus, we analyzed 76 samples (21 female, 52 first-episode unmedicated DPD, who were not treated with any mediation). The detailed information on patients is shown in Table 2.

**Figure 1:**
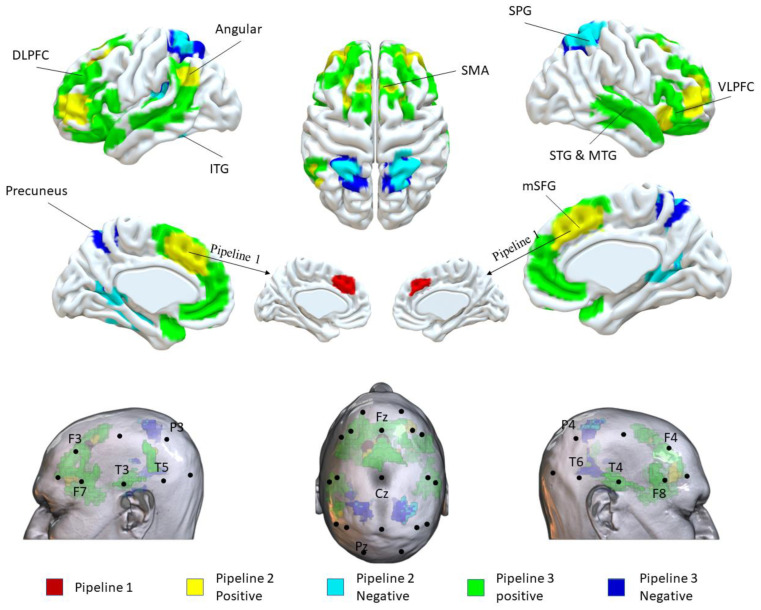
Potential targets for NIBS.

In pipeline 1, the medial superior frontal gyrus (mSFG) was identified, located on Fz in the 10–20 system locations. In pipeline 2, the bilateral dorsolateral prefrontal cortex (DLPFC; F3, F4), medial prefrontal cortex (mPFC; Fz), right ventrolateral prefrontal cortex (VLPFC; F8), and left angular (P3) were positively correlated with the DPD network. The bilateral precuneus (Cz-Pz,), superior parietal gyrus (superior to P4/P3), left inferior temporal gyrus (ITG, T3), and superior temporal gyrus (STG, T3) were negatively correlated with the DPD network. In pipeline 3, the bilateral mSFG (Fz), DLPFC (F3, F4), VLPFC (F7, F8), supramarginal gyrus (P3), supplementary motor area (SMA), middle temporal gyrus (MTG), and STG (T3, T4) were positively correlated with the largest number of DPD ROIs. The bilateral superior parietal gyrus (SPG, superior to P3/P4), precuneus (Cz-Pz), left ITG (T3), and STG (superior to T5) were negatively correlated with the largest number of DPD ROIs (Table 3 and Figure 1).

**Figure 2:**
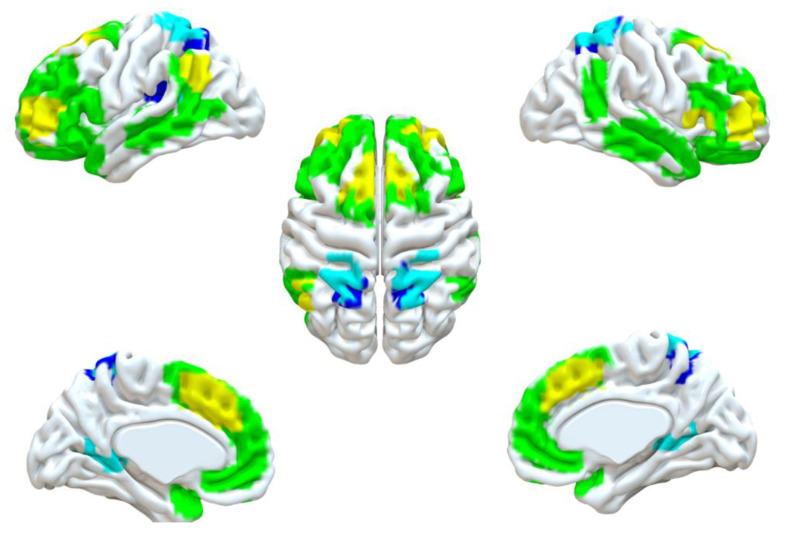
The results of sensitivity analyses (excluding treated patients).

Sensitivity analyses excluding treated patients showed similar results (Figure 2), which showed the findings were robust. In detail, the brain regions identified from pipeline 2 (positive and negative) and pipeline 3 (negative) were mainly congruent in the sensitivity analysis, except for the cluster size. One more result, the right angular, was added to the sensitivity analysis of pipeline 3 positive, and the rest of the results remained the same.

## 4. Discussion

An initial objective of this study was to find the potential NIBS targets to treat DPD. Stimulating mSFG, especially medial prefrontal cortex (mPFC), may be the most beneficial, as we identified in three pipelines. Bilateral DLPFC, precuneus, SPG, STG, SMG, and right VLPFC also could be potential targets.

The mPFC is the key brain region found in this study. The mPFC has been found to be activated differently in DPD patients from healthy controls with the tasks about self-recognition [37], emotional memory [38], emotional experience, and awareness of self [39]. In detail, Ketay et al. [37] found that DPD patients (*n* = 9) exhibited a greater activation than healthy controls (*n* = 10) in response to viewing their faces vs. that of a stranger in the frontal cortices (anterior cingulate cortex, bilateral mPFC, and left middle frontal gyri). A study by Medford et al. [38] indicated lower activation in bilateral frontal areas, bilateral precuneus, and cerebellum during target word recognition tasks in DPD patients (*n* = 10) than healthy controls (*n* = 12). Reference [39] examined brain hyper-activation in response to viewing aversive images in DPD patients (*n* = 14) as compared to healthy controls (*n* = 25) in the areas of the right lateral prefrontal cortex, bilateral primary visual cortex, bilateral anterior cingulate cortex (ACC), and left medial prefrontal cortex. Thus, according to previous studies, DPD is a disorder with the abnormality in mPFC. However, there is a lack of evidence to find its correlation with the symptoms in patients with DPD.

Regrettably, we found no NIBS on DPD trials targeting the mPFC. Considering the function of the mPFC and its effect on other mental diseases might help us propose strategies for targeting it to treat DPD, the mPFC has been proposed to serve as a variety of social, affective, and self-processes [40]. Healthy subjects who received mPFC TMS showed successful modulation of self-reference processing [41,42]. The mPFC also appears to be of transdiagnostic significance in multiple categories of psychiatric disorders [43]. A previous study suggested that mPFC rTMS treatment was beneficial and safe for psychiatric disorders, such as obsessive-compulsive disorder, substance use disorder, and major depressive disorder [44]. Patients with post-traumatic stress disorder can benefit from a combination of script-driven exposure with repeated deep TMS of the mPFC on fear extinction [45]. In alcoholics, rTMS directed to the mPFC significantly reduced blood cortisol levels and prolactinoma, suggesting an increase in dopamine [46]. Thus, we inferred targeting mPFC could improve symptoms of DPD patients, especially on “unreality of self” and “emotional numbing” aspects.

Bilateral DLPFC, SPG, STG, and right VLPFC could also be potential sites for DPD NIBS treatment, which were identified in the FC analysis. Several preliminary TMS studies have shown a beneficial effect on DLPFC [13,47], right temporo-parietal junction (TPJ) [25,48,49], and right VLPFC [50,51], while SPG, SMG, and precuneus still require further investigation.

Targeting DLPFC may help, but more parameters still need to be explored. Some authors presented a case [13] concerning a patient with DPD and depression comorbidity (CDS score 149, Montogmery–Asberg Depression Rating Scale (MADRS): 12) who began treating depressive and depersonalization symptoms using 1 Hz TMS stimulation applied to the right DLPFC. After session 7, the patient’s mood appeared to have improved, but depersonalization symptoms persisted (CDS:132, MADRS:4). The depersonalization symptoms continued to improve after the treatment switched to 10 Hz (32 sessions; CDS: 48; MADRS: 3). A similar case [47] (male with DPD and depression comorbidity) was treated with 20 Hz rTMS targeting left DLPFC, which improved depersonalization symptoms (CDS score from 175 to 126) in the patient. There were only two cases in which stimulation of the DLPFC was beneficial, and it remains to be explored whether low-frequency stimulation or high-frequency stimulation would be beneficial.

Targeting the right TPJ could be better documented. A case [25] described a male DPD patient with a CDS score of 96, treated via once-daily sections (on weekdays) of rTMS (1 Hz) on the right TPJ (location: TP4) using a MagPro R30 magnetic stimulator (70-mm coil). The patient’s CDS score decreased as the treatment duration increased. Another open-label study showed [48,49] that low-frequency rTMS to the right TPJ may show therapeutic promise in DPD, and that the anomalous body experiences score might represent a robust predictor of response for the remaining symptoms. In detail, six patients were responders in 3 weeks, and five in 6 weeks. In this study, the responders were defined by a CDS total score reduction ≥ 25%. Notably, the right TPJ was not found directly in our study. The brain regions found in our study were slightly inferior to TP4, but they could be also altered if stimulated on TP4.

Targeting the right VLPFC may also help. There was a significant reduction in depersonalization symptoms in patients after a single session of 1 Hz rTMS to the right VLPFC or TPJ stimulated during a small sample of random control trials (DPD/Healthy: VLPFC vs. TPJ = 8/11 vs. 9/9) [50]. Specifically, five of eight patients in the right VLPFC group were partial responders; four of nine patients in the right TPJ group were partial responders, and one was a full responder. Overall, 20 sessions of rTMS treatment to right VLPFC (*n* = 7) significantly reduced scores on the CDS by on average 44% (range 2–83.5%) with two full responders, four partial responders, and one non-responder [51]. The partial responder means a 25% reduction in CDS-state score, whereas the full responder means a 50% reduction. The CDS-state uses the mean score expressed as a percentage to assess “here and now” rates of patients.

Prior studies did not use sham stimulation, although placebo response was low among DPD patients [4]. Other NIBS techniques research on DPD patients was lacking. Further research is required to investigate the effect of the above sites.

We discovered the SPG as a potential target for the first time. SPG is a brain region associated with cognition, such as working memory [52,53], selective attention [54], and visuospatial attention [55]. The cognition of DPD patients was found impairment according to previous research [7,8,56]. It seems that targeting SPG could improve cognition in DPD patients, although future studies need to be conducted.

There are several limitations to this study. First, only five studies were included in the analysis, although the ALE method is robust. The limited studies included might result in bias of ROIs. Second, we found several targets for NIBS, but the target is just one of the factors influencing the effect. NIBS techniques, parameters, and the number of stimulation sessions could also influence the effect. NIBS techniques differ considerably and have complicated mechanisms for intervening in DPD. When using different NIBS techniques (e.g., tDCS, TMS) or different parameters, it may have a different effect even when targeting the same sites. It may be helpful to use biophysical modeling software, such as the works [57,58,59] done by the Danish Research Centre for Magnetic Resonance and the Technical University of Denmark (i.e., simNIBS [60] software). However, the application and optimization of various NIBS techniques are not the focus of this paper. Future studies will investigate the effects and mechanisms of NIBS techniques on intervening DPD Thus, future studies are needed. Finally, we do not know if the brain regions we identified are excitatory or inhibitory, which is important for some NIBS techniques, such as rTMS and tDCS.

## 5. Conclusions

In conclusion, our study identified several potential targets for NIBS treatment of DPD patients. The bilateral mPFC, DLPFC, SPG, STG, and right VLPFC could be potential sites. These findings may serve as a basis for future NIBS research and applications on DPD patients.

## Figures and Tables

**Table 1:** ALE analysis results.

ID	x	y	z	ALE	P	Z	Label	BA
1	−1	16	35	9.25 × 10^−3^	1.93 × 10^−5^	4.12	CG-L	32
2	−1	25	41	7.04 × 10^−3^	1.73 × 10^−4^	3.58	mFG-L	8
3	−1	25	35	6.82 × 10^−3^	2.39 × 10^−4^	3.49	mFG-L	9
4	−1	15	25	6.63 × 10^−3^	3.05 × 10^−4^	3.43	AC-L	33
5	−18	19	38	6.33 × 10^−3^	4.41 × 10^−4^	3.33	mFG-L	6
6	−1	30	22	4.97 × 10^−3^	2.78 × 10^−3^	2.77	AC-L	32

Abbreviation: L, left; CG, cingulate gyrus; mFG, medial frontal gyrus; AC, anterior cingulate; BA, Brodmann area.

**Table 2:** Information on patients.

	Untreated (*n* = 52)	Treated (*n* = 24)
Gender (Male/Female)	38/14	17/7
Age	23.9 ± 5.2	23.5 ± 6.7
Education	13.6 ± 2.6	13.9 ± 3.1
Duration (Years)	5.5 ± 4.8	4.5 ± 4.7
CDS total score	162.6 ± 45.5	180.9 ± 58.1
Numbing	31.5 ± 14.2	35.7 ± 16.4
Unreality of Self	40.0 ± 15.4	40.1 ± 14.0
Perceptual alterations	20.1 ± 12.7	20.8 ± 10.9
Unreality of surroundings	14.1 ± 5.6	16.0 ± 5.0
Temporal disintegration	21.0 ± 9.6	23.3 ± 10.7

**Table 3:** Potential targets identified from three pipelines.

			Peak Coordinates				
Cluster	Cluster Size	Peak Intensity	x	y	z	Brain Regions	10–20 System Locations
Pipeline 1							
1	572	0.0084	0	24	38	mSFG	Fz
Pipeline 2 Positive							
1	1098	30.0245	0	24	38	mSFG	Fz
2	544	21.024	−32	48	0	SFG and MFG_L	F3
3	360	20.0082	34	48	8	MFG_R	F4
4	73	16.9293	−54	−58	30	Angular_L	P3
5	54	20.9628	36	22	−12	IFG_R	F8
Pipeline 2 Negative							
1	316	−9.75294	30	−52	12	Precuneus_R	Cz-Pz
2	299	−6.66981	36	−48	62	SPG_R	Superior to P4
3	142	−8.70127	−20	−46	2	Precuneus_L	Cz-Pz
4	118	−6.55238	−38	−40	−16	ITG_L	T3
5	58	−4.51933	−30	−48	66	SPG_L	Superior to P3
6	52	−7.962	−36	−32	20	STG_L	T3
Pipeline 3 Positive							
1	1389	6	-	-	-	IFG_L	F7
1	499	6	-	-	-	mSFG_L	Fz
1	371	6	-	-	-	SFG_R	F4
1	359	6	-	-	-	MFG_R	F4
2	1494	6	-	-	-	IFG_R	F8
3	919	6	-	-	-	STG_R	T4
4	640	6	-	-	-	MTG_L	T3
5	502	6	-	-	-	SMG	P3
6	780	6	-	-	-	SFG_L	Fz
6	697	6	-	-	-	SMA_L	Fz-Cz
6	321	6	-	-	-	SMA_R	Fz-Cz
Pipeline 3 Negative							
1	683	5	-	-	-	SPG_R	Superior to P4
2	505	5	-	-	-	SPG_L	Superior to P3
3	300	6	-	-	-	Precuneus_R	Cz-Pz
4	130	6	-	-	-	Precuneus_L	Cz-Pz
5	93	6	-	-	-	ITG_L	T5
6	52	6	-	-	-	STG_L	Superior to T3

## Data Availability

The data presented in this study are available on request from the corresponding author.

## Supplementary Materials

The following supporting information can be downloaded at: https://www.mdpi.com/article/10.3390/brainsci12081112/s1. Figure S1 PRISMA flowcharts; Figure S2 Brain surface mask; Table S1 information of included studies. References [30,31,32] are cited in the supplementary materials.
