# Supplementary material for: Potential Targets for Noninvasive Brain Stimulation on Depersonalization-Derealization Disorder
Source: Brain Sci. 2022 Aug 21;12(8):1112. doi: 10.3390/brainsci12081112 (PMC9406113; doi:10.3390/brainsci12081112)
Supplement: Supplementary file 1 [file brainsci-12-01112-s001.zip › brainsci-1841062-supplementary.pdf]

Supplemental Materials

1. Detailed information of meta-analysis

1.1 Methods

This systematic review adhered to PRISMA 2020 guidelines and followed a predetermined published protocol.

1.1.1 Search strategy

To gather the articles for this review, we searched PubMed, Web of Science, PsycINFO, Embase, and the Cochrane Library from inception to September 2021. The named databases were searched using combinations of the two key concepts from MeSH, namely: depersonalization and Magnetic Resonance Imaging. The within-concept subject headings and keywords were combined with the "OR" command, while the two strings of searches were combined with the "AND" command in each database. Furthermore, we hand-searched the references sections of the identified studies, as well as those of relevant review articles. Key authors were contacted to identify whether any further trials could be identified and seek additional data from studies as required.

1.1.2 Inclusion and Exclusion Criteria

For the purpose to identify the potential mechanisms of depersonalization–derealization disorder (DPD), we set the following inclusion and exclusion criteria to filtrate neuroimage studies focus on the DPD patients compared with healthy controls.

Inclusion criteria were studies with patients assessed with at least one diagnostic basis for depersonalization disorder, including ICD or DSM diagnostic standard, DES, CDS or other standardized rating scales, diagnosis of specific clinical institution or physician, or description of related symptoms. Exclusion criteria were studies solely on geriatric or adolescent populations, or participants with depersonalization secondary to organic brain disorders, or

symptoms of depersonalization with other diseases as the main diagnosis, such as epilepsy, depression, anxiety, or substance abuse. For on the basis that depersonalization experienced would have a different etiology and be managed differently to the focus of this review. The studies focused on patients diagnosed as other dissociative disorder were such as dissociative identity disorder and dissociative amnesia. All kinds of neuroimage studies were included. It was excluded if the study did not report the coordinates of contrast clusters.

1.1.3 Study Selection

After a summary of retrieved articles, results with the same author and similar titles were confirmed by reading the full text, only one of the two articles with the same content would be retained. All the rest were reviewed by the lead authors (ZS and WS) who read study titles to remove any non-relevant articles based on the inclusion and exclusion criteria of each of this review listed above. The remaining abstracts were read and judged as relevant, possibly relevant, or not relevant to this search based on their specific inclusion and exclusion criteria. All reviews/studies deemed to be possibly relevant were read in full and independently judged against inclusion and exclusion criteria by two reviewers (WS and ZS). Disputes were resolved by consensus and consultation when necessary with a third reviewer (SM). Hand searches of the references from all the included reviews/studies were conducted to identify studies missed in the bibliographic searches. Relevant reviews/studies were processed as detailed above. The corresponding authors were contacted to request additional information if needed.

1.1.4 Data Extraction, Synthesis, and Presentation

We extracted the coordinates of peak activation for each cluster from the published studies that satisfied all inclusion and exclusion criteria. In detail, the coordinates of the cluster compared DPD patients and healthy control were extracted.Most studies reported the Talairach coordinates, so if the coordinates were reported in MNI space, they were converted to Talairach coordinates using the Lancaster transform. Then, all Talairach coordinates were entered into a database of active clusters that were used as input for the meta-analysis. ALE

meta-analysis was performed via GingerALE (cluster-level FWE = 0.01, threshold permutation=1000, P value =0.001). In order to perform the next step (functional connectivity), we transformed the Talairach map to MNI map via Data Processing and Analysis for Brain Imaging (DPABI) version 5.1 (http://rfmri.org/dpabi)[1].

1.2 Results

1.2.1 PRISMA flowcharts

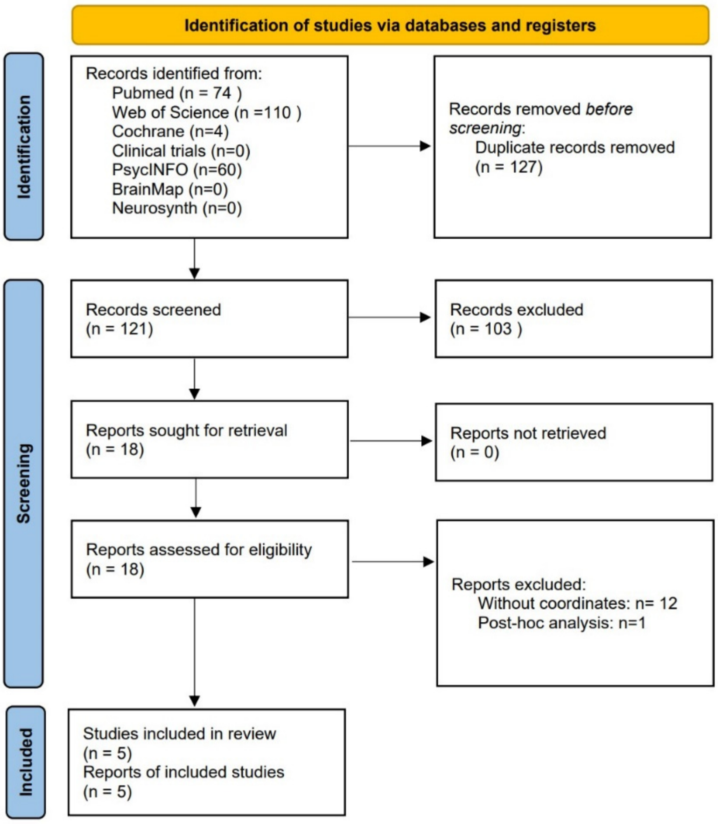

Figure S1 PRISMA flowcharts[2]

1.2.2 the information of included studies

Table S1 information of included studies

Author	Year	Sample Size (DPD/HC)	Female	Age	Diagnostic Criteria	Task
Phillips et al	2001	6/6	1/2	28.3(22-34)/33.8(24-48)	PSE >2 ; DES ≥ 13	emotional stimuli task
Lemche et al	2016	9/12	4/5	36.11 ± 2.34/27.25 ± 1.95	DSM-IV; CDS > 70	facial expression stimuli task
Medford et al	2016	14/25	3/11	33.7±8.9/29.8±5.68		emotional stimuli task
Medford et al	2006	10/12	1/0	31.2±9.3/27.8± 3.6	DSM-IV	emotional stimuli task
Ketay et al	2014	9/10	-/-	33.2±11.2/31.9± 11.8	SCID-D	self and stranger facial stimuli task
Lemche et al	2007	9/12	4/5	36.11 ± 2.34/27.25 ± 1.95	DSM-IV; CDS > 70	facial expression stimuli task

PSE, Present State Examination; DES, Dissociative Experience Scale; CDS, Cambridge Depersonalization Scale; DSM, Diagnositc and Statistical Manul of Mental Disorders

2. Detail information of identifying potential targets methods

2.1 Brain surface mask

The mask included the following brain regions: the bilateral pre- and post-central gyrus; superior and middle frontal gyrus; superior, inferior, and middle occipital gyrus; superior and inferior parietal lobule; supramarginal gyrus; angular gyrus; superior temporal gyrus; superior temporal pole; middle temporal gyrus (MTG); middle temporal pole; inferior temporal gyrus; opercular inferior frontal gyrus (IFG); Rolandic operculum; triangular IFG; superior medial frontal gyrus; calcarine sulcus; orbital middle, superior and inferior frontal gyri; orbital medial frontal gyrus; supplementary motor area (SMA); paracentral lobule; precuneus; and cuneus.

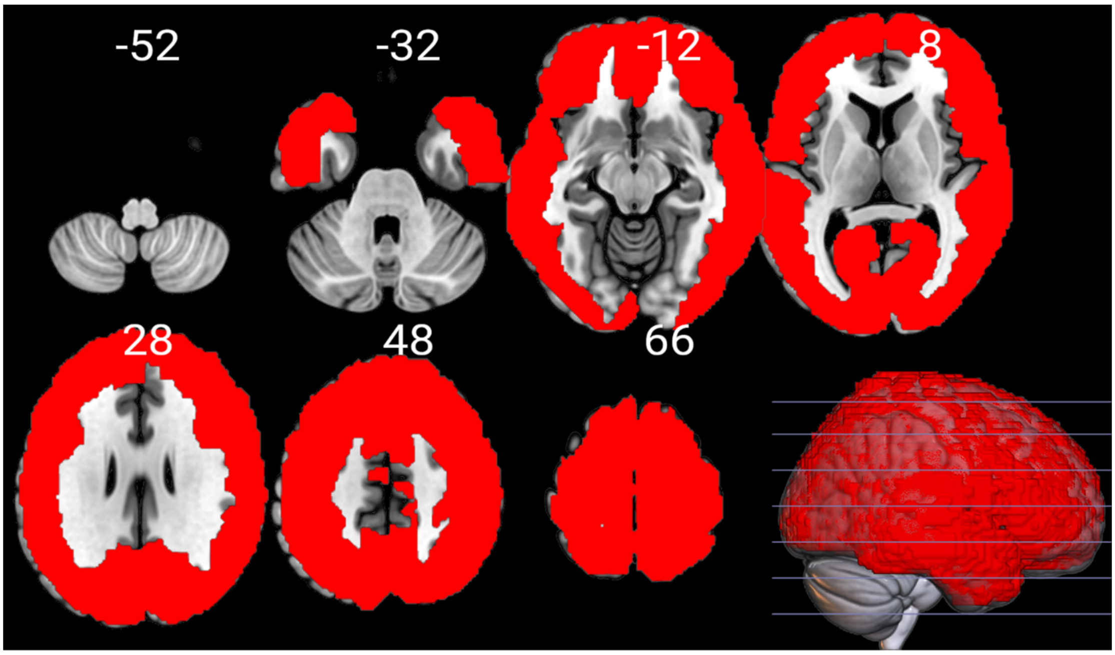

Figure S2 Brain surface mask

2.2 MRI data acquisition

Rs-fMRI were acquired with a single-shot, gradient-recalled echo-planar imaging sequence with the following parameter: repetition time=2000ms, echo time = 30 ms, flip angle = 90°, matrix = 64×64, field of view = 200 mm × 200 mm, slice thickness = 3.5 mm, gap = 1 mm, 33 axial sections, and 240 volumes.

High-resolution brain structural images were acquired with a T1-weighted three-dimensional (3D) multi-echo magnetization-prepared rapid gradient-echo (MPRAGE) sequence (echo time: 3.39 ms, repetition time: 2530 ms, slice thickness 1.3 mm, voxel size: 1.3 × 1 × 1 mm^3, field of view (FOV): 256 × 256 mm^2 and volume number: 128).

2.1.3 Image preprocessing

All the image processing was conducted under the DPABI and Statistical Parametric Mapping (SPM) 12 (www.fil.ion.ucl.ac.uk/spm/)[3]. We removed the first 10 volumes of rs5-fMRI data. Further, those with a maximum head motion of 3 mm or more and a relative root

mean square (RMS) of 0.2 mm or more were excluded from consideration. The default preprocessing pipeline of DPABISF was used, including both functional and structural steps.

Briefly, the preprocessing contained slice-time corrected, realigned, normalized ($4 \times 4 \times 4$ mm^3 in MNI space) and smoothed ($4 \times 4 \times 4$ mm^3). Outliers (> 3 SD and > 0.5 mm) were detected by the Artifact Detection Tool (www.nitrc.org/projects/artifact_detect/). Structure images were segmented into gray matter (GM), white matter (WM), and cerebral spinal fluid (CSF), and normalized to MNI space. Then, linear regression using WM and CSF signals (CompCor; 10 components for WM and five components for CSF), linear trend, subject motion (six rotation/translation motion parameters and six first-order temporal derivatives), and outliers (scrubbing) was conducted to remove confounding effects. A band-pass filter (0.01-0.1 Hz) is then applied to the residual blood oxygen level dependent (BOLD) time series.

Reference

1. Yan C-G, Wang X-D, Zuo X-N, Zang Y-F. DPABI: Data Processing & Analysis for (Resting-State) Brain Imaging. Neuroinformatics. 2016;14:339–351.
2. Mj P, Je M, Pm B, I B, Tc H, Cd M, et al. The PRISMA 2020 statement: an updated guideline for reporting systematic reviews. BMJ. 2021;372:n71.
3. Ashburner J. SPM: A history. NeuroImage. 2012;62:791–800.
